# Workplace trauma and professional quality of Life in clinical and forensic psychiatry: the CRITIC study

**DOI:** 10.3389/fpsyt.2024.1228335

**Published:** 2024-03-01

**Authors:** Anthony F. T. Bloemendaal, Astrid M. Kamperman, Annette E. Bonebakker, N. Kool, M. Olff, C. L. Mulder

**Affiliations:** ^1^ Department of Psychiatry, Erasmus Medical Centre, Rotterdam, Netherlands; ^2^ Dual Disorder Treatment Centre, Fivoor, The Hague, Netherlands; ^3^ Amsterdam University Medical Centre (UMC), Department of Psychiatry, University of Amsterdam, Amsterdam Neuroscience and Amsterdam Public Health & ARQ National Psychotrauma Centre, Diemen, Netherlands; ^4^ Antes Psychiatric Care, Parnassia Group, Rotterdam, Netherlands

**Keywords:** aggression, personal life history, quality of life, compassion fatigue, clinical psychiatry, frontline staff

## Abstract

**Background:**

Frontline staff in psychiatry need to perform at a very high professional level in order to ensure patient and community safety. At the same time they are exposed to high levels of stress and workplace trauma. This may have severe consequences for their professional quality of life. In addition, health care workers in general have higher incidence levels of childhood adversity than the general population. The CRITIC (CRITical Incidents and aggression in Caregivers) Study aims to improve increased understanding of the interaction between personal life history (childhood adversity and benevolence), individual capabilities, exposure to trauma and violence at work and Professional Quality of Life (ProQOL).

**Method:**

The Critic Study is a cross-sectional survey of these aspects in frontline, treatment and administrative staff in clinical and forensic psychiatry. We aim to include 360 participants. Participants will be asked to complete questionnaires on childhood adversity and childhood benevolence (assessing personal life history), professional quality of life, current trauma and violence exposure, current mental health (depression, anxiety and stress), coping, social support, work engagement and resilience. In this study we will examine the moderating role of adverse and benevolent childhood experiences in the association between workplace trauma exposure and professional quality of life. Finally, a theoretical model on the relationships between trauma, stress and coping in the context of professional functioning will be tested using structural equation modelling.

**Discussion:**

The CRITIC study examines which factors influence the complex relationship between childhood adversity and benevolence, and ProQOL in healthcare workers. It also aims to provide insight into the complex relationship between personal life history, individual characteristics, exposure to trauma and violence at work and ProQOL. The results can be used for designing interventions to increase resilience to trauma and to improve professional quality of life among health care professionals.

**Trial registration:**

The CRITIC study has been approved by the Medical Ethical Committee of the Erasmus Medical Centre, under trial registration number NL73417.078.20

## Background

### Frontline staff and workplace violence

Exposure to violent, stressful and traumatic events at work poses a major problem for workers and health care providers (1). The prevalence of these forms of workplace trauma exposure in psychiatric care has been studied since the last years of the previous century. Newman (2), Hilton (3) and Babiarrcyk et al. (4), provide us with an overview of studies done around the world, presenting high percentages (>70%) of nurses working in acute and inpatient psychiatry experiencing violence (verbal and physical) at work. Two other recent studies describe similar results: Itzhaki et al. (5) reported in 2018 that almost 89% of mental health nurses working in acute and inpatient psychiatry in Israel experienced verbal aggression in the last year and 56% physical violence. Niu et al. (6) reported rates that were very similar (82% verbal aggression and 56% physical aggression) in acute inpatient psychiatry in Taiwan in the previous year.

A systematic review by Mento et al. (7), showed health care professionals across the whole span of health care encounter workplace violence. Psychiatric departments, emergency services and geriatric units ranked very high on verbal and psychological abuse, but also physical and even sexual abuse occur frequently (7).

These aggressive incidents can have severe consequences. Schablon, Wendeler et al. (8) found in 2018 that between 27% and 44% of nurses felt high levels of self-reported stress because of the incidents. This survey was conducted in Germany amongst nurses working in inpatient psychiatry, geriatric care, other residential facilities and day care centres (N=1984), of whom 94% had experienced verbal abuse and 70% physical abuse (8). Additionally, three systematic reviews reported severe consequences of workplace violence, including decreased physical, psychological and emotional functioning and various aspects of impaired worker performance (quality of care, financial, social and general impact) (7, 9, 10).

Ielapi et al. (11) found that nursing staff in psychiatry – who work in close contact with patients – were 4 times more likely to encounter aggression, compared to other (treatment) staff. In their systematic review, Liu et al. (12) underlined this fact and included other settings as well, consistently showing the group with most exposure to workplace violence are nursing staff, psychiatrics and physicians in psychiatry and emergency departments (12). In this study we will use the term “frontline staff” to describe all people working in the direct and 24 hour care of admitted patients.

### Professional quality of life and compassion fatigue

Quality of life is severely impacted by violent, stressful and traumatic experiences (13). The concept of Professional Quality of Life has been introduced Larsen and Stamm (14) describing the complex relationship between positive and negative variables at the individual, organizational, and societal levels that influence the well-being and effectiveness of the health care professional. The dynamic interaction of positive and negative factors creates this overarching construct of professional quality of life (14, 15).

Compassion is an important factor within the concept of Professional Quality of Life. Compassion fatigue (CF) describes the process when health care professionals gradually desensitize to patient stories, the quality of care declines, errors increase and there are higher rates of depression, anxiety and stress (16). Conceptually, CF has been defined as comprising of two central and clinical features: (1) secondary trauma, and (2) job burnout. In 2020 the CF concept has been validated in a systematic review and meta-analysis by Cavanagh et al. (17), although they did not hypothesize which individuals are more likely to develop CF than others. The concepts of compassion fatigue and Professional Quality of Life are still used widely as shown in this systematic review (18).

### Influence of adverse and benevolent childhood experiences

Another relevant aspect that might influence the Professional Quality of Life, is the personal life history of health care workers.

It is a well-known fact that psychiatric patients have relatively high levels of trauma and neglect in childhood (19). In recent years, attention has also been drawn to professionals working in mental health care, who also reported higher incidence levels of childhood adversity than the general population (20) and then professionals working in other areas (20–22).

Two recent systematic reviews support the notion that mental health professionals have experienced more adversity in childhood. Bryce et al. (23) report finding consistently high percentages of ACEs in therapists and mental health professionals. In an even more elaborate systematic review, Mercer et al. (20) report percentages ranging from 25% to 31% of mental health professionals reporting 4 or more ACEs, compared to 12,5% in the original ACE study in 1998 by Felitti (24).

To date however, little research is known to the authors about the prevalence of adverse childhood experiences (ACEs) in frontline staff, treatment staff and non-clinical staff in general and forensic in- and outpatient psychiatry and its impact on ProQOL. A smaller study by Bouchard and Rainbow (25) reported a negative relation between ACE and ProQOL in Doctor of Nursing Practice students. Mercer et al. (26) showed that staff working with people with intellectual disabilities who report more adverse childhood experiences also experience more burn-out and secondary traumatic stress.

In addition, there is increasing interest into the counterpart of adverse childhood experiences (ACEs): benevolent childhood experiences (BCEs) (27). It is believed that higher BCEs are protective against the long-term effects of ACE´s and that this is associated with resilience, less trauma related symptomatology and less stress exposure during pregnancy (27, 28). Little is known, however, about ACEs and BCEs in frontline staff, treatment staff and administrative workers, and how they interact in case of stressors at the workplace. Little is also known about the moderating effect of BCEs on the association between workplace violence and ProQOL. For this reason Merrick and Narayan (29) propose to structurally measure BCE alongside ACE measurements.

### Stress-coping model

**Figure 1:**
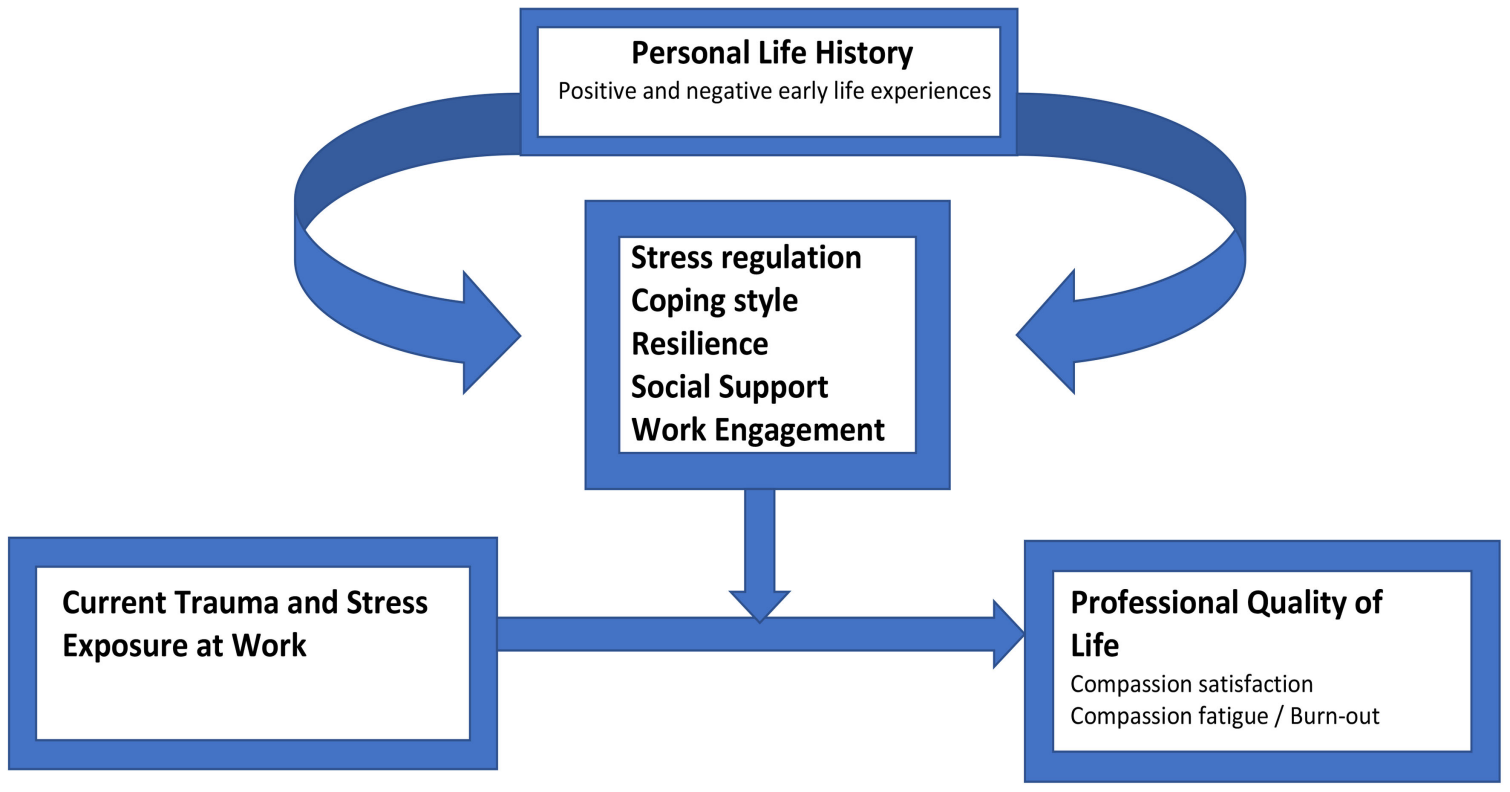
Theoretical concept of the CRITIC Study.

The CRITIC (**CRIT**ical **I**ncidents and aggression in **C**aregivers) study, developed and instigated by the authors, investigates the complex set of factors influencing Professional Quality of Life. The theoretical framework of the CRITIC study (**Figure 1**) elaborates on current stress-coping models as described below. Thus, inspired by the stress-coping-social support model (30, 31), it may be that factors including coping styles and the presence of social support also have a mediating or sometimes moderating effect on the association between ACEs, workplace trauma exposure and professional quality of life. Therefore, variables related to these concepts will also be included in this study.

### Theoretical model

A hypothetical theoretical model for the complex relationship between trauma, coping and ProQOL was inspired by the model proposed by Olff, Draaijer, Langeland and Gersons (32). This model, still being used in the context of gender differences (33), combines the extensive literature on trauma and stress with contextual information (gender, personal life history, coping style, social support), thus making this model more comprehensive than other models on coping and stress. Three recent reviews show most research has focused on the direct relation between coping and stress, or include only a small number of other factors (34–36) The CRITIC Study hopes to expand on Olff et al.’s model (32) and include several other factors, most notably childhood adversity and benevolence, that may be of importance in understanding the complex relationships between personal life history (childhood trauma), coping, workplace trauma exposure and ProQoL. The research model is depicted in **Figure 1**.

In summary, the topics addressed above indicate the importance of gaining more knowledge about the associations between workplace trauma exposure and professional quality of life in frontline and treatment staff, and how this association is moderated by ACE´s and BCE´s. The following aims of this study have been conceived to address this gap in our understanding.

## Objectives

### Primary objective

This study aims at answering the following research questions:

The primary research question has two parts:

1. What is the extent of Adverse and Benevolent Childhood Experiences in frontline staff and treatment staff in general and forensic psychiatry in comparison to non-clinical staff?
2. To what extent is the relation between workplace trauma exposure and ProQol moderated by childhood adversity and benevolence?

The secondary research questions are:

1. What is the overall level of Professional Quality of Life (ProQoL) in health care staff compared to the control group of non-clinically working health care staff?
2. To what extent is the association between workplace trauma exposure and professional quality of life mediated by stress, coping and social support?
3. To what extent is the association between workplace trauma exposure and professional quality of life mediated by psychological symptoms?

## Materials and methods

This is an explorative study using a cross-sectional design using self-administered fully structured questionnaires.

## Study population

360 participants (see sample size calculation) will be recruited from inpatient psychiatric and forensic wards of mental health organizations, mainly situated in the Western part of the Netherlands.

In these organisations, also non-clinically working staff, including administrative staff will be approached to participate, as a comparison group.

### Inclusion criteria

In order to be eligible to participate in this study, a subject must meet all of the following criteria:

- Work in clinical or forensic psychiatry in The Netherlands as frontline staff, treatment staff or non-clinical staff.
- Have enough mastery of the Dutch language to complete the measurements.
- Have given informed consent.
- Over the age of 18.

### Exclusion criterium

Not providing informed consent will be an exclusion criterium.

### Variables

Demographic information (age, gender, marital status), work history and workplace characteristics will be collected.

The following questionnaires will be used:

#### Professional quality of life

Professional Quality of Life is measured with the ProQoL (37). This is a self-report measure developed to assess compassion satisfaction and compassion fatigue, which has two subscales: secondary traumatic stress and burnout. The ProQOL contains 30 items, equally divided over the (sub)scales, that are rated on a 5 point scale, ranging from 1 (never) to 5 (very often). Stamm (37) reports that the ProQOL has demonstrated good construct validity and inter-scale correlations. Cronbach’s alpha reliabilities were 0.75 (burnout subscale), 0.81 (secondary traumatic stress subscale) and 0.88 (compassion satisfaction scale). As shown by their systematic review, the ProQoL questionnaire is still widely used (18).

#### Adverse childhood experiences

The Adverse Childhood Experiences – International Questionnaire (ACE-IQ) and Adverse Adult Experiences – International Questionnaire (AAE-IQ) are developed by the World Health Organisation (38) to measure the incidence of adverse experiences in childhood (ACE-IQ) and in adulthood (AAE-IQ). Each questionnaire consists of 20 items, scored yes or no. Every yes is 1 point, adding up all the yes scores gives the ACE total score. In most studies, a total ACE score of 4 or higher is seen as a high risk factor for developing physical, social and psychological problems and the score is dichotomised in two groups (ACE score < 4 and ACE score 4 or higher) (39). This questionnaire has been translated to Dutch by Van der Feltz-Cornelis (40) and is currently being validated in the Dutch population. The English version shows good reliability (Cronbach’s alpha = 0,85) (41).

The Child Trauma Questionnaire (42) assesses trauma and neglect in childhood. It contains 5 subscales: physical abuse, emotional abuse, sexual abuse, physical neglect and emotional neglect. The 28 items are scored on a 5 point Likert scale. Reliability is high (Cronbach’s alpha = 0,95 for the total scale) (42). This questionnaire has been validated and translated to Dutch by Arntz and Wessel (43). A recent meta-analysis shows its continued relevance (44).

#### Benevolent childhood experiences

An adaptation of the ACE Questionnaire by Felitti et al. (24) inquires about Benevolent Childhood Experiences. The BCE questionnaire consists of 10 items and was developed and validated by Narayan et al. (27). In their study they identified three groups: High BCE – Low ACE, High BCE – High ACE and Low BCE – High ACE. For each group, high was more than 4 ACE’s or BCE’s on average (27). The BCE has shown good reliability and validity (CFI = 0,94; NFI = 0,92) (45). The questionnaire was translated into Dutch by the authors of this manuscript.

#### Workplace trauma exposure

Workplace trauma exposure will be assessed using the Perception of Prevalence of Aggression Scale (POPAS). It measures 15 types of disruptive and aggressive behaviour on the workfloor in the past year. Also, it measures frequency and impact of these experiences on the staff. It has been validated in English in 2005 (46) and in Dutch in 2001 (47) and shows good psychometric quality. The POPAS has recently been used and validated in Turkish (48), for instance.

#### Traumatic events

The Life Events Checklist for DSM-5 (LEC-5) (49) is a self-report measure designed to screen for potentially traumatic events in a respondent’s lifetime. The LEC-5 assesses exposure to 16 events known to potentially result in PTSD or distress and includes one additional item assessing any other extraordinarily stressful event not captured in the first 16 items. It has been translated and validated in Dutch by Boeschoten et al. (50) showing adequate validity. Recent use of this questionnaire shows its relevance and reliability for research purposes (51).

The LEC-5 is intended to gather information about the potentially traumatic experiences a person has experienced. There is no formal scoring protocol or interpretation per se, other than identifying whether a person has experienced one or more of the events listed. Respondents indicate varying levels of exposure to each type of potentially traumatic event included on a 6-point nominal scale, and respondents may endorse multiple levels of exposure to the same trauma type. The LEC-5 does not yield a total score or composite score (49). In a recent study it has been validated and shows good reliability (52).

Current posttraumatic stress symptoms are measured with the PCL-5 (53). The PCL-5 is a 20-item self-report measure that assesses the 20 DSM-5 symptoms of PTSD (53); Dutch translation is done by Boeschoten et al. (50). Overall, results indicate that the PCL‐5 is a psychometrically sound measure of PTSD symptoms (Cronbach’s alpha = 0,94) (53), which has recently been validated in a sample of veterans (54).

#### Depression, anxiety and stress symptoms

This will be measured with the Depression Anxiety and Stress Scale (DASS) (55). The short 21 item form is used in this study. It has three subscales, with 7 items each: depression, anxiety and stress. It has been translated to Dutch and validated within the Dutch population by (56). A recent study confirmed its validity (57).

#### Coping Style

Coping style is measured with the Dutch Brief Coping Strategy Indicator (DUBRISCI), a brief version of the Coping Strategies Indicator (CSI) (58, 59). The questionnaire consists of 9 items (3-point scale), to assess three basic modes of coping: problem solving, seeking social support, or avoiding the event (range 0–18). The CSI has been found to be valid and reliable (58) and is still being used in research (60).

#### Social support

Social Support is measured with the Social Support List – 6 (SSL-6), a questionnaire which has been validated in English (61) and Dutch (62), showing good psychometric quality. It contains 6 questions with an a and b question, totalling 12 items. The instrument consists of three subscales: ‘everyday social support’, ‘social support in problem situations’ and ‘esteem support’. Recent studies show its value for research purposes (63, 64).

#### Resilience

Resilience is measured with the Resilience Evaluation Scale (RES), a 9-item questionnaire. Subscales are self-confidence and self-efficacy. It has been translated in Dutch and validated, showing good psychometric quality (65). This instrument has recently been translated and validated in Chinese (66) and Indonesian (67). Also it was incorporated in a recent systematic review of measures of resilience (68).

#### Work engagement

Work Engagement is measured using the UWES (Utrechtse Work Engagement Scale), a 15 item self-report scale which reliably and validly measures the concept of work engagement (69, 70).

### Statistical analysis

For frontline staff, treatment staff and non-clinical staff we will report the proportion reporting unique ACE and BCE, as well as the total number of ACEs and BCEs. Proportions and counts will be reported including their 95% confidence interval. Differences between frontline staff, treatment staff and non-clinical staff will be estimated using logistic regression models for dichotomous outcomes, and Poisson regression models for count data.

Associations between independent and dependent variables will be estimated using regression analyses. Depending on the measurement level of the outcome variable, we will employ a linear, logistic or multinomial regression model. Moderation and mediation of predictor variables will be tested using hierarchical regression analyses in line with the protocols set forward by Baron and Kenny in 1986 (71), shown to be one of the most used protocols for this type of analysis (72). After establishment of mediation and/or moderation of a predictor variable, the analysis will be repeated adjusting for confounding variables. To control for false positive results, we will employ the Benjamini-Hochberg procedure, developed in 1995 (73) and still used widely in research (74).

Next, the theoretical model will be tested using structural equation analysis (SEM) (75). The major advantage of this analysis is the ability to simultaneously estimate direct and indirect effects between multiple independent and dependent variables. To allow for multivariate non-normality of the variables and missing data-points, we will estimate the path coefficients between unique variables, and the fit of the full model, using robust maximum likelihood estimation (76). Fit of nested models will be compared using a Chi2-test with Satorra-Bentler correction (77), which is still widely used today (78).

Data will be analyzed using SPSS 28. SEM analysis will be conducted using MPlus 8.

### Sample size calculation

Based on previous research (24, 79–81) we expect a small to moderate difference between frontline staff, treatment staff and non-clinical staff with respect to the prevalence of one or more ACEs. To show a significant (alpha = 5%; beta = 80%) difference between the three subgroups we will need a sample size of 360 participants in total.

Moderation analyses will be tested using 8 tests. To decrease the false discovery rate (FDR), we will use the Benjamini-Hochberg procedure (73). FDR acceptance rate (Q) will be set to 10%. The moderating effect of ACEs on the relation between workplace trauma and professional quality of life will be studied within the subgroups of frontline staff and clinical staff participants.

Thus, to show a significant (B-H corrected alpha = 1.25%; beta = 80%) small sized (f2 = 0.05) moderating effect of a single predictor variable we will need a sample size of 240 participants. This sample size will also enable us to detect a small to moderate effect size (f2 = 0.10) in a multiple regression analysis as well as show significant paths with a moderate effect size (r=0.4) in a model containing 12 observed variables, and 2 latent variables using structural equation modelling analysis.

## Discussion

This study explores the associations between childhood adversity and benevolence, coping and professional quality of life in health care professionals in psychiatry. The results can be used for designing interventions to increase resilience to trauma and to improve professional quality of life among health care professionals. This is also important because workplace violence is a major problem with serious consequences, leading to reduced professional quality of life, job turnover and sick leave (82). We need to understand how to improve retention of staff by helping them overcome any personal issues hampering their job satisfaction or making them vulnerable to stress related problems. We also hope to better understand the associations between compassion fatigue and the experience of different traumatic events.

### Potential clinical implications

With the results of this study, we will be better equipped to help frontline staff avoid burn-out, secondary trauma and compassion fatigue. We will be able to predict who needs what kind of support in dealing with workplace violence and stress. The quality of care will improve as the teams will be more stable and the individuals will receive support tailored to their specific need.

### Limitations

Potential pitfalls are the inherent bias of self-report questionnaires and the fact that for all participants the early childhood is long ago as the minimum age in our inclusion criteria is 18 years old. It is a known fact that memories of (early) childhood are potentially distorted.

The current cross-sectional design does not allow for testing causal relationships and following participants for a longer period of time and tracking fluctuations in mental health symptoms, exposure to workplace violence, other life events and professional quality of life.

### Strengths

The strength of this study is using a theoretical model describing the associations between variables under investigation in this study. In addition, we will have a large number of participants (N = 360). We also have included non-clinical staff in order to test whether experiencing workplace trauma is specifically related to frontline staff. We included a broad range of variables, measured with validated questionnaires. Finally, this is the first time a study has aimed to understand the relationship between personal life history and professional quality of life in mental health care, including frontline staff.

### Conclusions

This study investigates the little understood relation between personal life history, workplace exposure to violence, coping, and professional quality of life in frontline mental health care workers and administrative staff in clinical and forensic psychiatry.

## Ethics statement

Ethics approval has been granted by the MREC of the Erasmus Medical Centre in Rotterdam. Trial registration number is NL73417.078.20.

## Author contributions

AFB and AK wrote the manuscript. AEB, NK, CM and MO read and corrected the manuscript. All authors contributed to the article and approved the submitted version.
